# Renal Fibrosis mRNA Classifier: Validation in Experimental Lithium-Induced Interstitial Fibrosis in the Rat Kidney

**DOI:** 10.1371/journal.pone.0168240

**Published:** 2016-12-21

**Authors:** Hans-Peter Marti, Aaron Jeffs, Andreas Scherer, John Leader, Catherine Leader, Jennifer Bedford, Robert Walker

**Affiliations:** 1 Department of Clinical Medicine, University of Bergen, Bergen, Norway and Haukeland University Hospital, Bergen, Norway; 2 Department of Biochemistry, University of Otago, Dunedin, New Zealand; 3 Spheromics Inc., Kontiolahti, Finland; 4 Department of Medicine, University of Otago, Dunedin, New Zealand; The University of Manchester, UNITED KINGDOM

## Abstract

Accurate diagnosis of fibrosis is of paramount clinical importance. A human fibrosis classifier based on metzincins and related genes (MARGS) was described previously. In this investigation, expression changes of MARGS genes were explored and evaluated to examine whether the MARGS-based algorithm has any diagnostic value in a rat model of lithium nephropathy. Male Wistar rats (n = 12) were divided into 2 groups (n = 6). One group was given a diet containing lithium (40 mmol/kg food for 7 days, followed by 60mmol/kg food for the rest of the experimental period), while a control group (n = 6) was fed a normal diet. After six months, animals were sacrificed and the renal cortex and medulla of both kidneys removed for analysis. Gene expression changes were analysed using 24 GeneChip^®^ Affymetrix Rat Exon 1.0 ST arrays. Statistically relevant genes (p-value<0.05, fold change>1.5, t-test) were further examined. Matrix metalloproteinase-2 (MMP2), CD44, and nephroblastoma overexpressed gene (NOV) were overexpressed in the medulla and cortex of lithium-fed rats compared to the control group. TGFβ2 was overrepresented in the cortex of lithium-fed animals 1.5-fold, and 1.3-fold in the medulla of the same animals. In Gene Set Enrichment Analysis (GSEA), both the medulla and cortex of lithium-fed animals showed an enrichment of the MARGS, TGFβ network, and extracellular matrix (ECM) gene sets, while the cortex expression signature was enriched in additional fibrosis-related-genes and the medulla was also enriched in immune response pathways. Importantly, the MARGS-based fibrosis classifier was able to classify all samples correctly. Immunohistochemistry and qPCR confirmed the up-regulation of NOV, CD44, and TGFβ2. The MARGS classifier represents a cross-organ and cross-species classifier of fibrotic conditions and may help to design a test to diagnose and to monitor fibrosis. The results also provide evidence for a common pathway in the pathogenesis of fibrosis.

## Introduction

Glomerulosclerosis and tubulointerstitial fibrosis represent the final stage of most chronic kidney diseases (CKD) regardless of their underlying origin [1]. Renal fibrosis is a progressive disorder ultimately leading to end-stage kidney disease (ESKD), characterized by proliferation and transformation of fibroblasts into myofibroblasts, deposition of fibronectin and collagens I and III into the interstitium, monocyte/macrophage, and T-cell infiltration, microvascular rarefaction, and podocyte depletion. [2].

Metzincins and related genes (MARGS) play important roles in ECM remodeling in fibrotic conditions. Metzincins are zinc-dependent metalloproteases that can be subdivided into those which contain a disintegrin and metalloprotease domain (ADAM), those with a disintegrin and metalloprotease domain with thrombospondin-motif (ADAMTS), and also serralysins, papalysins and matrix metalloproteases (MMP) with their tissue inhibitors (TIMP) [3, 4]. A transcriptomic classifier consisting of 19 MARGS, discriminating human renal allograft biopsies with or without interstitial fibrosis/tubular atrophy (IF/TA), has been previously defined [3]. Subsequently, the analysis was extended to non-transplant solid organs with and without fibrosis [5], aged rodents [6] and renal injury following ureteral obstruction [7].

In this investigation, gene expression was examined in a rat model to investigate whether the previously described MARGS based fibrosis classifier had diagnostic value in an experimental rat model of lithium-induced nephrogenic diabetes insipidus (NDI) which over the subsequent months of lithium treatment showed progressive focal renal fibrosis [8]. After 6 months, urine production in the control group was46 ± 9 μL/min/kg body weight and in the lithium group was 449 ± 43 μL/min/kg body weight (P< 0.001), typical of NDI. Plasma lithium concentration in the experimental group was maintained within the therapeutic dosage recommended for humans (at 6 months; lithium group, 0.9 ± 0.1 mM/L) [8]. Plasma electrolytes, plasma osmotic pressure were comparable in both groups, and there was no evidence of deterioration in renal function, although there was a slight increase in proteinuria in the lithium-treated animals [8]. Some data were verified by immunohistochemical staining and quantitative PCR (qPCR) amplification.

## Materials and Methods

### Animals

Rat experiments were performed as described previously [8, 9]. Male Wistar rats weighing 200 g were obtained from the Hercus-Taieri Resource Unit, University of Otago, Dunedin, New Zealand. Ethical approval was provided by the Animal Ethics Committee from the University of Otago (92/08), according to New Zealand National Animal Welfare guidelines.

The rats were divided into two groups, an experimental group (n = 6), that received dietary lithium for 6 months, and a control group (n = 6). The control group received a standard rodent diet (Specialty Foods, Perth, Australia) and tap water *ad libitum* for 6 months. The experimental group was fed 40 mM lithium/ kg dry food for the first week, followed by 60 mM lithium/kg dry food for a further 23 weeks. It has been shown that this is adequate to raise and maintain plasma lithium levels to between 0.8–1.3 mM/L, equivalent to a therapeutic dose in human patients [8]. Water was available *ad libitum*. All rats given lithium were supplied with a salt block to maintain sodium balance and prevent lithium intoxication. Body weight and water intake were measured every second day. After 6 months, the animals were sacrificed by decapitation and their kidneys removed. Blood samples were taken immediately, the plasma separated by centrifugation and stored at -20°C. The two kidneys from each animal were cut in half longitudinally; one half was put in 10% neutral buffered formalin for histology, and the other 3 halves were snap frozen in liquid nitrogen and stored at -80°C until further use.

### Preparation of RNA and hybridization of microarrays

Portions of the medulla and cortex were removed from the frozen kidneys after partial thawing, and homogenised in Trizol^R^ (Invitrogen, Life Technologies, ThermoFisher Scientific, Waltham, MA, USA). RNA was extracted by precipitation with ethanol, followed by cleaning with Norgen Total RNA columns (Norgen Biotek Corp., Canada). RNA was quantified using a ND-1000 spectrophotometer (NanoDrop Technologies, Wilmington, DE, USA) and its (purity and) quality determined on a Bioanalyzer 2100 (Agilent Technologies, Santa Clara, CA, USA). Only RNA samples with an integrity number>8.0 were used. 50 ng total RNA was processed by using a GeneChip WT Terminal Labeling Kit (Affymetrix, Santa Clara, CA, USA) and hybridised to Affymetrix Rat Gene 1.0 ST gene chips. Chips were stained and washed with an Affymetrix GeneChip Fluidics Station 450, and scanned in an Affymetrix GeneChip 7G Plus scanner. All procedures were done according to the manufacturer's instructions. 4 groups of arrays were used: control medulla (6), lithium medulla (6), control cortex (6) and lithium cortex. (6)

### Analyses of microarray data

CEL files from the 24 GeneChip^®^ Rat Exon 1.0 ST arrays (Affymetrix, Santa Clara, CA, USA) probe sets were subjected to RMA background correction, quantile normalization, log base 2 transformation, and median polish, using Partek Genomics Suite v 6.5 (www.Partek.com). For both cortex and medulla datasets, the effect of the technical factor “Scan date” was stronger than the effect “Treatment” which was the actual biological effect which the analysis should focus on. The technical effect was eliminated by ANOVA, including “Treatment” and “Scan Date” as factors. Following batch effect adjustment, exon probe sets were summarized into gene IDs by calculating the mean of the exon probe sets. This resulted in 28,894 transcript IDs. Expression filters were applied, which included only those features with log2-expression levels of at least 6 in at least 33% (i.e. 4 of 12) samples in a dataset. 2-way ANOVA was performed for cortex and medulla separately. For further gene ontology or pathway analysis, a significance p-value threshold of 0.05, and a minimum absolute fold change of 1.5 were arbitrarily chosen, attempting to enable future cross-platform validation [10]. Hierarchical cluster analysis was performed with standardized normalized signal intensity values, applying Euclidean distance, and Ward’s method.

Data are available in the repository Gene Expression Omnibus, http://www.ncbi.nlm.nih.gov/geo/query/acc.cgi?acc=GSE83610.

### Gene set enrichment analysis (GSEA)

GSEA was performed using the Broad Institute Tool (http://www.broadinstitute.org/gsea) along with GSEA statistics (obtained from the GSEA website, http://www.broadinstitute.org/cancer/software/gsea/wiki/index.php/Main_Page). Briefly, GSEA determines the degree of enrichment of members of gene sets at ends of a fold-change ranked gene list. The enrichment score reflects the degree of enrichment of a gene set at one end of the ranked gene list. The normalized enrichment score is the enrichment score normalized to the size of the gene set. Gene sets are defined by prior knowledge, e.g. on biological pathways or gene ontologies. For the analysis of Gene Ontology, a set of 1454 gene sets was used (external URL: http://amigo.geneontology.org), for the analysis of pathways, 901 gene sets were applied. These comprised 216 sets from BIOCARTA, 186 KEGG sets, 430 sets from REACTOME, 10 sets from SA (Sigma Aldrich), 8 from SIG (Signaling Gateway), 28 sets from ST (Signaling Transduction KE), 20 sets from SA Biosciences, and the MARGS set. The 20 SA Biosciences (SAB) genesets were: cAMP, EMT, Extracellular Matrix, Fibrosis, Chemokines, Cytokines_common, Hypoxia, Inflammatory cytokines, nephrotoxicity, TGFβ, VEGF, WNT, T and B cell activation, TNF, Wound healing, TH17, MAPK, GPCR, Growth factors, and NFκB.

The analysis was performed with gene symbols, applying permutation on gene sets, and a random seed 100. There was no size limitation for the genesets.

Statistical relevance was achieved when the absolute normalized enrichment score was larger than 1.5 and false discovery rate was smaller than 0.05.

### Classifier analysis

Classification of the sets of 12 samples was performed as described previously [3]. The algorithm was linear discrimination analysis. To avoid overfitting, the number of variables in a model was limited to 11 from the group of 15 of the 19 human MARGS genes present on the rat array, internal validation was performed by the leave-one-out cross-validation method. The variables in the models were automatically selected by the software by optimization of the results.

### Histology and immunohistochemistry

For histological and immunohistochemical studies, kidneys were wax embedded and sections cut at 3 μm. For histological examination, sections were stained in Masson’s Trichrome, using conventional protocols. Sections were examined on an Olympus Provis AX70 microscope and photographed with a Spot digital camera.

For immunohistochemical treatments, sections were dewaxed and rehydrated, antigen retrieval (microwaving in 10 mM/L citrate buffer, pH 6.0) was carried out, and endogenous peroxidase activity was blocked with 3% H_2_O_2_ in phosphate buffered saline (PBS). After preincubation in 1% bovine serum albumin (BSA, Sigma-Aldrich, St Louis, MO, USA) in PBS to block nonspecific binding, sections were labelled with the appropriate primary antibody. Antibodies used were against transforming growth factor-2 (TGFβ2, polyclonal, rabbit, sc-90, Santa Cruz Biotechnology, Dallas, TX, USA), CD44 (mouse, 554869, Pharmingen, BD Biosciences, USA)), and nephroblastoma over-expressed gene (NOV/CCN3, polyclonal, goat, AF1640, R & D Systems, Minneapolis, MN, USA). Labelling of the tissue was visualized using a horseradish peroxidase-coupled secondary antibody (goat anti-rabbit or rabbit anti-goat immunoglobulins (IgG, DAKO, Glostrup, Denmark), followed by incubation with diaminobenzidine (Sigma-Aldrich, St Louis, MO, USA). After dehydration and clearing, sections were mounted in DPX and viewed on an Olympus AX70 Provis microscope. Representative regions were digitally recorded using a Spot camera. Negative controls were carried out either by omitting the primary antibodies or by using appropriate blocking peptides, and positive controls were used against chosen tissues.

To better interpret the immunohistochemical picture from TGFβ2, images were analysed using ImageJ. Using Adobe Photoshop, a series of 20 images were collected from 10 sections, 5 control and 5 from lithium-treated kidneys, by randomly traversing cortical and outer medullary regions. The resulting images were viewed using ImageJ, and the percentage of brown pixels in a 100 μ square mask determined, after colour deconvolution and thresholding.

### qPCR

The RNA samples from the medulla and cortex, used for the microarrays, were reverse transcribed using a Superscript III reverse transcriptase single strand DNA synthesis kit (Invitrogen, Life Technologies, Thermo Fisher Scientific, Waltham, MA, USA) with oligo-dT primers. The resulting cDNA was then used with Assays—on-Demand TaqMan amplicon probes, Mastermix (Applied Biosystems, Thermo Fisher Scientific, Waltham, MA, USA) and the FAM reporter dye. Standard cycling conditions were used on an Applied Biosystems 7700 analyser. Assays-on-Demand TaqMan probes used were for TGFβ2, CD44, and NOV/CCN3 and the housekeeping gene β-actin. Relative quantification was carried out using the ΔC_t_ method.

## Results

### Histology of lithium-induced renal fibrosis

**Fig 1:**
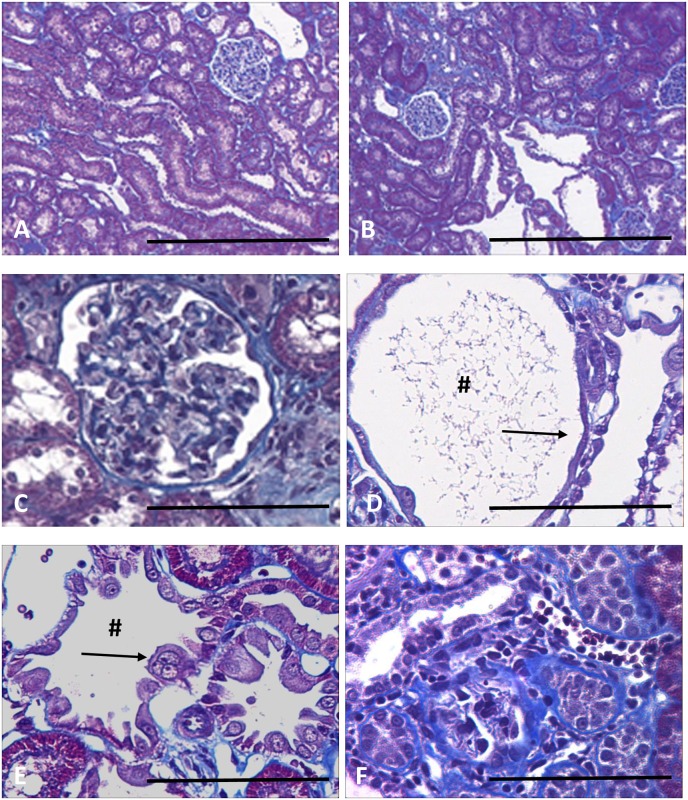
Sections of rat kidney stained with Masson’s Trichrome. Low power sections of normal rat kidney (A) and rat fed a lithium diet for six months (B), in which collecting duct tubules show cyst-like dilatations. The lithium treated rats show glomerulosclerosis (C, arrow), collecting duct dilations # (C, D), and epithelial linings either greatly attenuated (D, arrow) or with enlarged cells, often appearing multinucleate (E, arrow). The cortex has areas of focal fibrosis(F). Scale bar, A, B, 200 microns; C-F, 50 microns.

After 6 months on a lithium-fed diet, rats exhibited a striking focal tubulointerstitial fibrosis (Fig 1A and 1B), characterized by increased interstitial collagen deposition, increased numbers of interstitial myofibroblasts, and grossly dilated collecting ducts, with abnormal cytology. These abnormalities are illustrated at higher magnification in Fig 1A–1D. Many glomeruli show evidence of a focal glomerulosclerosis (Fig 1C, arrow), while others show attachments to the capsular wall. Cortical collecting ducts are frequently greatly dilated (Fig 1D), and their epithelial lining ranges from attenuated cells spread thinly over the surface (Fig 1D) to greatly enlarged cells intruding into the lumen (Fig 1E), some of which appear to be multinucleate (Fig 1E, arrow). Deposition of extracellular matrix, usually adjacent to collecting ducts, compressed or obliterated the lumens of the enveloped tubules (Fig 1F). In spite of these obvious histological changes, there was no significant deterioration in renal function.

### cDNA microarray analysis

Gene expression changes of these 163 MARGS were analysed in the cortex and medulla of lithium-treated rats and compared to those of control rats. Genes with a p-value<0.05 and an absolute fold change >1.5 were considered to be differentially expressed. These are illustrated in Tables 1 and 2.

**Table 1:** MARGS expression changes in the renal cortex between control and lithium-treated rats.

Transcript Cluster ID	Gene Symbol	RefSeq	p-value	Control group mean signal intensity [log2]	Lithium group mean signal intensity [log2]	Fold-Change (lithium vs. control)
**10896541**	**NOV**	**NM_030868**	**1.5E-05**	**4.78**	**7.16**	**5.19**
**10809540**	**MMP2**	**NM_031054**	**1.4E-04**	**6.81**	**8.60**	**3.45**
**10847761**	**CD44**	**NM_012924**	**2.4E-04**	**5.33**	**6.56**	**2.36**
10834670	ADAMTSL2	ENSRNOT00000036995	1.4E-03	7.38	6.95	-1.35
**10768642**	**LAMC2**	**NM_001100640**	**2.1E-03**	**5.41**	**6.25**	**1.79**
10770577	TGFB2	NM_031131	2.2E-03	6.23	6.77	1.46
10740869	TNFRSF12A	NM_181086	4.0E-03	6.35	6.89	1.45
10812021	ITGB1	NM_017022	4.5E-03	8.36	8.69	1.26
10884080	LAMB1	NM_001106721	1.1E-02	7.95	8.10	1.11
10809136	MMP15	NM_001106168	1.7E-02	7.18	7.55	1.29
10864185	ADAMTS9	NM_001107877	1.8E-02	6.10	6.33	1.18
10925859	LAMA1	NM_001108237	1.8E-02	6.56	6.26	-1.23
10785063	BMP1	NM_031323	2.4E-02	6.49	6.84	1.28
**10761047**	**SERPINE1**	**NM_012620**	**3.5E-02**	**5.78**	**6.56**	**1.72**
10715787	NFKB2	NM_001008349	3.6E-02	6.74	7.02	1.22
10909482	HYOU1	NM_138867	4.3E-02	7.45	7.64	1.14
10833985	ADAMTSL4	NM_001034012	4.6E-02	6.65	7.15	1.41

MARGS with p-value<0.05 and |FC|>1.5 are in bold. Groupwise mean signal intensity is expressed as log2 value. Fold change is calculated as 2^(log2(Mean (lithium))-log2(Mean (control))).

**Table 2:** MARGS expression changes in the renal medulla between control and lithium-treated rats.

Transcript Cluster ID	Gene Symbol	RefSeq	p-value	Control group mean signal intensity [log2]	Lithium group mean signal intensity [log2]	Fold-Change (lithium vs. control)
**10896541**	**NOV**	**NM_030868**	**1.5E-07**	**5.91**	**9.91**	**16.00**
**10809540**	**MMP2**	**NM_031054**	**7.2E-05**	**7.48**	**9.65**	**4.51**
**10847761**	**CD44**	**NM_012924**	**8.5E-04**	**6.97**	**8.11**	**2.21**
**10785063**	**BMP1**	**NM_031323**	**8.5E-04**	**6.79**	**7.50**	**1.64**
**10929396**	**COL4A4**	**NM_001008332**	**3.0E-03**	**8.82**	**8.12**	**-1.63**
**10924719**	**COL4A3**	**NM_001135759**	**5.1E-03**	**9.46**	**8.84**	**-1.53**
10885251	HIF1A	NM_024359	5.4E-03	9.52	9.02	-1.41
10787856	TLL1	NM_001106081	6.9E-03	5.80	6.39	1.50
**10901231**	**TIMP3**	**NM_012886**	**8.4E-03**	**11.86**	**11.15**	**-1.63**
10740843	MMP25	ENSRNOT00000046876	8.8E-03	5.66	6.09	1.34
10874282	TNFRSF25	NM_001137644	1.1E-02	6.24	6.58	1.27
10715787	NFKB2	NM_001008349	1.1E-02	7.44	7.72	1.21
10931712	TNFSF14	XM_001059278	1.1E-02	5.79	6.07	1.21
10893863	ADAMTSL5	NM_001108071	1.1E-02	6.24	6.60	1.28
**10915933**	**ADAMTS15**	**NM_001106810**	**1.2E-02**	**6.33**	**6.96**	**1.55**
**10792421**	**PLAT**	**NM_013151**	**1.3E-02**	**10.04**	**8.85**	**-2.28**
10770577	TGFB2	NM_031131	1.5E-02	6.39	6.73	1.26
10733258	ADAMTS2	NM_001137622	1.6E-02	6.24	6.55	1.24
10812021	ITGB1	NM_017022	2.2E-02	9.47	9.14	-1.26
10715566	HIF1AN	NM_001113749	2.6E-02	7.51	7.23	-1.21
10737532	COL1A1	NM_053304	2.7E-02	7.71	8.27	1.48
10788899	ADAM9	NM_001014772	2.7E-02	8.59	8.22	-1.29
10937403	COL4A5	ENSRNOT00000025677	3.5E-02	7.60	7.11	-1.40
**10757726**	**ELN**	**NM_012722**	**4.1E-02**	**7.01**	**7.75**	**1.67**
**10752839**	**ADAMTS1**	**NM_024400**	**4.4E-02**	**6.60**	**7.46**	**1.81**
10791565	VEGFC	NM_053653	4.8E-02	6.32	6.58	1.19

MARGS with p-value<0.05 and |FC|>1.5 are in bold. Groupwise mean signal intensity is expressed as log2 value. Fold change is calculated as 2^(log2(Mean (lithium))-log2(Mean (control))).

**Fig 2:**
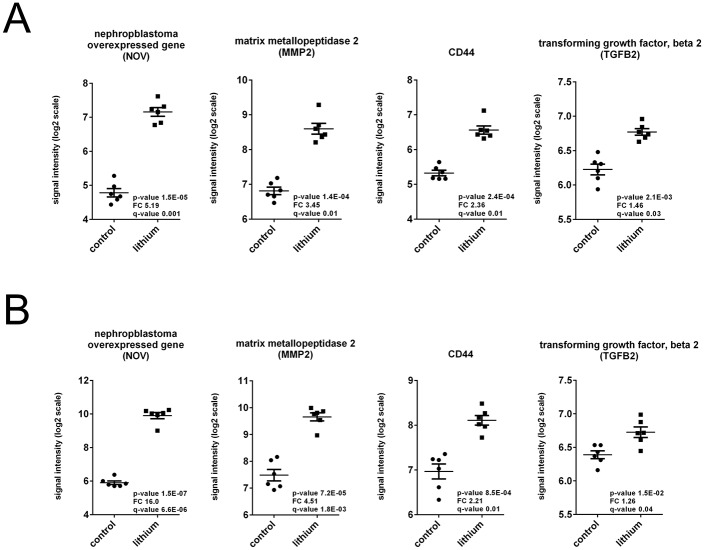
Scatter plots of signal intensities of NOV, TGFβ2, MMP2 and CD44 in the renal cortex (A) and medulla (B) from control rats and rats fed lithium for 6 months. Each point represents the signal intensity (a surrogate value of expression level) of mRNA in a sample. The expression differences between control and lithium-treated samples are clear (p values included).

**Fig 3:**
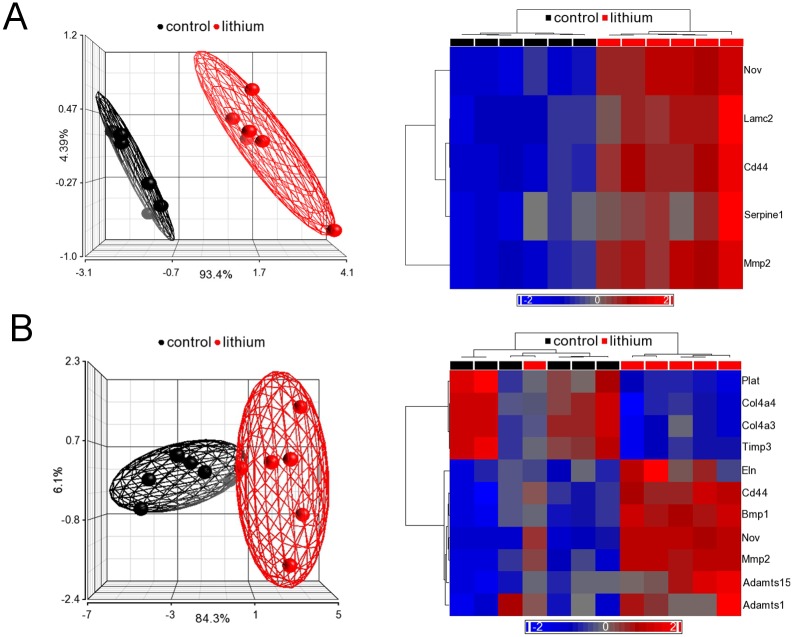
Principal component analysis (PCA) and hierarchical cluster analyses of MARGS in the renal cortex (A) and medulla (B) of kidneys from control and lithium-fed rats (6 months). **A.** PCA (left) and hierarchical cluster analysis (right) using 5 MARGS genes from cortex (expression filtered, p-value < 0.05, |FC| > 1.5); **B.** PCA (left) and hierarchical cluster analysis (right) using 11 MARGS genes from medulla (expression filtered, p-value < 0.05, |FC| > 1.5). MARGS are sufficient to separate the two groups. In PCA, each sphere represents one sample. The ellipsoids represent 95% of the data of a group. In both PCA plots in A and B, red indicates expression above median, blue indicates expression below median. The depth of the colour indicates extent of expression above or below median.

In the cortex dataset, 17 passed the p-value filter, 5 of those also passing the fold change filter, as shown in Table 1. In the medulla dataset, 26 transcripts had a p-value<0.05, of those 11 had an absolute fold change larger than 1.5 (Table 2). Interestingly, in both renal cortex and medulla, the 3 transcripts which responded most significantly to the lithium treatment were identical: NOV, MMP2, and CD44. Scatter plots in Fig 2 illustrate the expression differences for those genes as well as TGFβ2, another important molecule known to be involved in the development of fibrosis. In both datasets, differentially expressed MARGS transcripts led to a separation of control and lithium-treated samples in principal component analysis (PCA), and explained a large percentage of the variance between both sample groups in the first component (cortex: 96.8%, medulla: 89.6%), as depicted in Fig 3A and 3B. One lithium-treated sample in the medulla dataset did not cluster with the other lithium samples (Fig 3B). This is most likely due to biological variation or possibly the dissection of the tissue in the response to lithium treatment. Since the sample did not qualify as a technical outlier, it was left in the analysis.

### Classifier performance

To examine whether a MARGS-based composite classifier, previously identified in gene expression studies of human tissues [3], would be able to classify the rat fibrosis samples correctly, 15 of the19 MARGS genes of the classifier, which were represented on the rat GeneChip^®^ Affymetrix 1.0 ST microarray, were used in this study. Classification was performed as described previously: the algorithm “linear discrimination analysis”, with combinations of up to 11 genes for the classification of a set of 12 samples to avoid overfitting, and leave-one-out cross-validation, was used. Strikingly, for both cortex and medulla of the rat dataset, the classification rate using 11 MARGS genes was 100% (Table 3). The most frequently used genes were MGP, ADAMTSL3, and CD44, indicating their major contribution to classification models and their discriminative power.

**Table 3:** Fibrosis classifier showing 100% correct classification rate of Lithium versus Control (for explanation see text).

	medulla	Cortex
Number of genes	11	11
Lithium correct classification rate	6 (of 6)	6 (of 6)
Control correct classification rate	6 (of 6)	6 (of 6)
Correct classification rate (%)	100	100
Sensitivity (%)	100	100
Specificity (%)	100	100
Area under ROC	1	1

Classifier genes (n = 11). ADAMTS5, COL3A1, LAMB1, MGP, PAPLN, PLG, THBS2, THSD1, TNFSF10, TNSSF13B, VEGFA.

ADAMTS5, COL3A1, LAMB1, MGP, PAPLN, PLG, THBS2, THSD1, TNFSF10, TNSSF13B and VEGFA were able to classify all samples of medulla and cortex correctly with a classification rate, sensitivity and specificity of 100%.

### Gene set enrichment analyses (GSEA)

GSEA, focusing on 5 pathway gene sets known to be involved in fibrosis and ECM remodeling was used: MARGS, ECM, fibrosis, immune response (e.g. lymphocyte activation and T-cell activation) and TGFβ signaling [3, 5]. GSEA ranks transcripts by fold-change between two analysis groups and measures whether transcript pathway members are enriched at either end of the ranked list, assigning p-value. The association of genes to gene sets can then be used to calculate an enrichment score for those sets.

**Fig 4:**
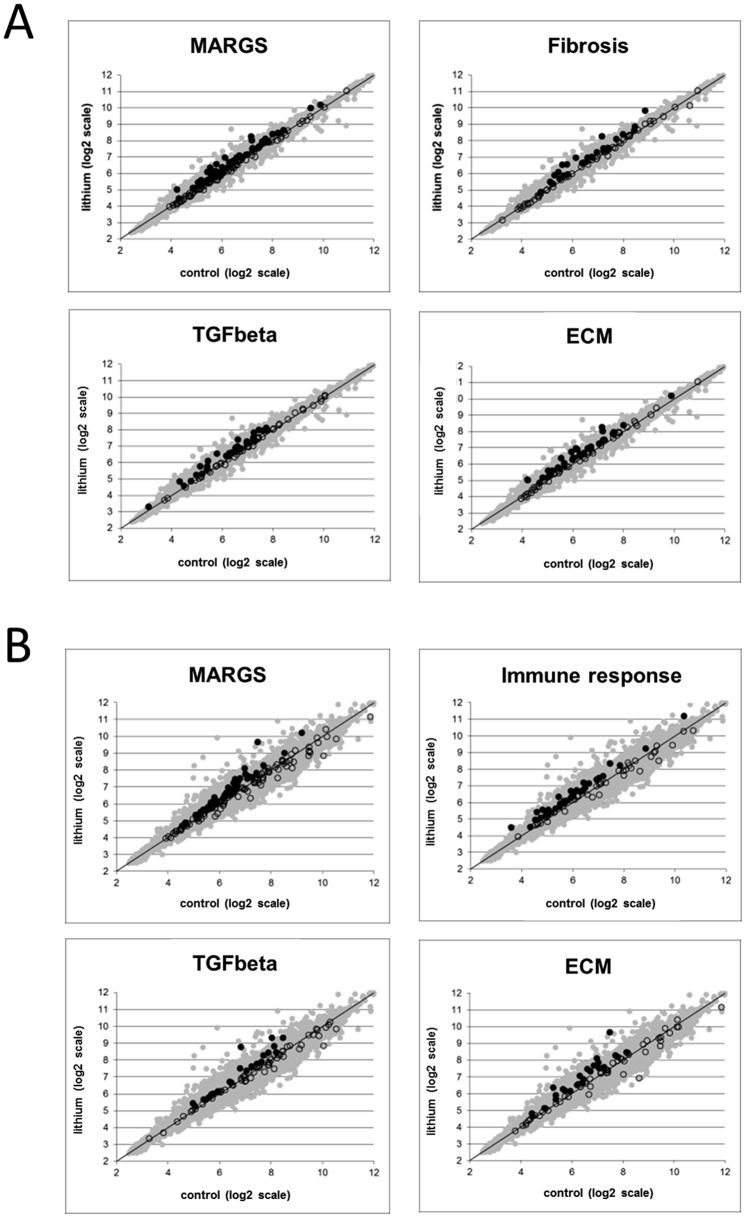
Gene Set Enrichment Analysis with microarray expression data from the renal cortex and medulla of rats fed lithium for 6 months. The mean signal intensity of a probe set (a surrogate value of expression level) across all lithium-fed samples (y-axis) is plotted against the mean signal intensity across all control samples (x-axis). Each point represents a single probe. **(A)** Gene sets with functional gene annotation enrichment for MARGS, fibrosis, TGFβ2 and ECM genes in the renal cortex; **(B)** Gene sets with functional gene annotation enrichment for MARGS, immune response, TGFβ2 and ECM genes in the medulla. With the exception of the MARGS gene set, all gene sets in this figure were extracted from www.SABiosciences.com.

GSEA plots illustrate the statistical enrichment of the gene sets in the cortex (Fig 4A) and medulla (Fig 4B) from control and lithium-treated rats. Each point represents the mean signal intensity (expression level) per group of a probe set within a gene set. No enrichment would be indicated by the position of a point along the identity line. Interestingly, the gene sets MARGS, fibrosis, TGFβ, and ECM are enriched in both lithium-treated cortex and medulla, but only the gene set “immune response” is enriched in medulla from lithium-treated rats, even to a more significant level than MARGS themselves (p<0.001, NES = 2.40, vs. p = 0.01, NES = 1.52, respectively).

### Immunohistochemistry and qPCR

To confirm the conclusions derived from the microarray data, further analytical studies were performed on selected gene products which showed significant changes following lithium treatment, such as TGFβ2, CD44, and NOV (CCN3) in renal cortical and medullary samples. TGFβ1 was also investigated, as a protein known to be involved in the fibrotic process, but did not show a significant change in the microarray data.

### NOV/CCN3

**Fig 5:**
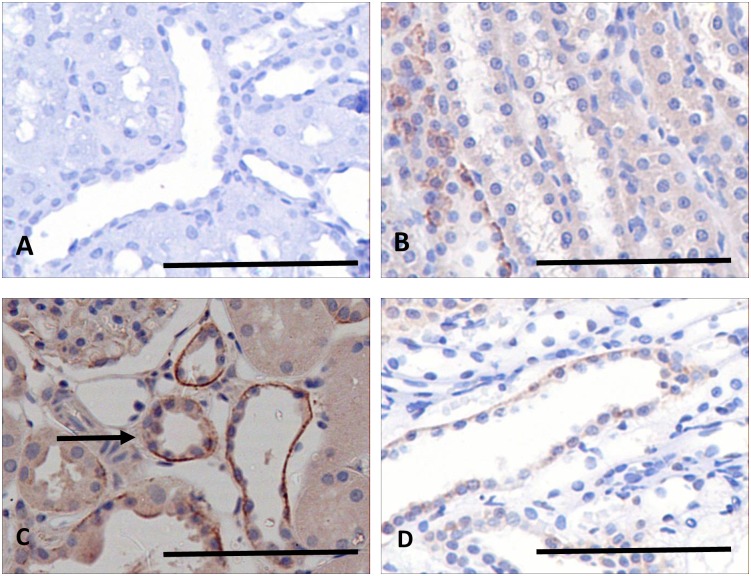
Sections of kidneys of control and lithium-treated rats probed for NOV/CCN3. In control rats the protein is apparently absent from the cortex (A), and barely detectable in the outer medulla (B), in basolateral margins of collecting ducts, but absent from thick ascending limbs. In contrast NOV occurs basolaterally in epithelial cells of the distal tubules and cortical collecting ducts of lithium-treated rats, (C, arrows) and weakly in collecting ducts of the outer medulla (D). Scale bar, 50 microns.

In marked contrast to the large changes in expression of NOV shown in the microarray data, immunohistochemistry of the protein appears to be weak (Fig 5A and 5B) and reveals that NOV is not detectable in the basolateral region of the cortical collecting ducts of the control animals but is barely detectable in the medullary collecting ducts of controls (Fig 5B). Exposure to lithium causes an upregulation of this expression in the cortex (Fig 5C) and which also extends into the outer medulla (Fig 5D) but its expression appears to be weak and confined here to cells of the collecting ducts.

The increase in expression of NOV induced by lithium exposure was also confirmed by quantitative PCR. Data obtained showed that there was an increase in NOV gene expression following six months of lithium exposure by more than a factor of 2 (2.79 ± 0.2; p> 0.005, n = 6) in the cortical extracts, and by 2.38 ± 0.23 (p>0.005; n = 6) in samples from the medulla.

### CD44

**Fig 6:**
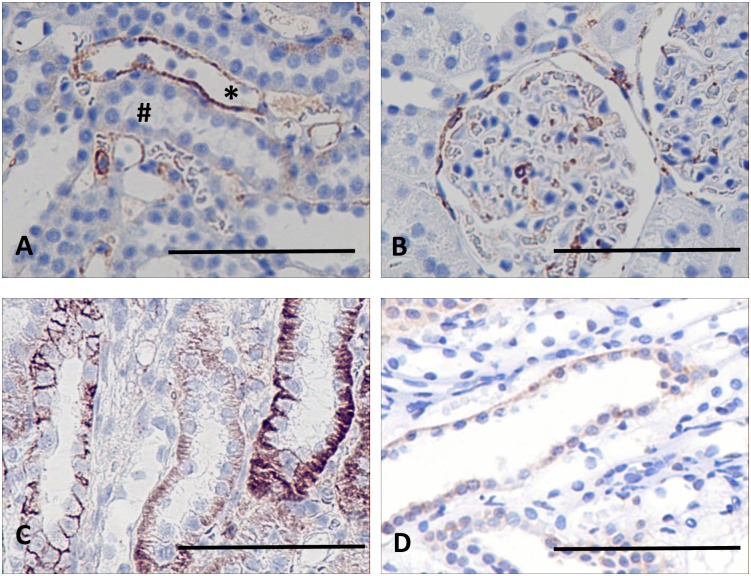
Sections of kidneys of control and lithium-treated rats probed for CD44. In control kidneys (A, B) there is strong labelling basolaterally in the distal tubule (A), and weak labelling in the glomeruli (B). The collecting ducts (A) are unstained. In the lithium-treated rats there is strong staining in both collecting ducts (C) and thick ascending limbs (C), while the staining of the glomeruli is unchanged (D). Scale bar, 50 microns.). Scale bar, 50 microns.

Immunohistochemistry of CD44 in the kidneys of control rats (Fig 6A) revealed its presence mainly in the basolateral membranes of distal tubules, although its occurrence was sporadic, and absent from the collecting ducts. It was also expressed in some epithelial cells lining Bowman’s capsule (Fig 6B). The label was also detected in the outer medullary collecting ducts, but only in some cells. In contrast, examination of the kidneys of lithium treated rats (Fig 6C and 6D) showed a substantial increase in expression. Although there was little change in the staining of the epithelial cells of Bowman’s capsule (Fig 6D), there was uniformly strong basolateral staining in the medullary collecting ducts and thick ascending limbs (Fig 6C), as well as the dilated cortical collecting ducts and distal tubules, and this strong expression was extended into the collecting ducts of the outer medulla.

qPCR of extracts of the cortical and medullary regions substantiated the qualitative impression given by immunohistochemistry and the microarray data. In the cortex of lithium-treated rats, abundance of CD44 mRNA increased by a factor of 1.17 ± 0.14; ns; n = 6), an insignificant change relative to the control animal. In the medulla, however, qPCR showed a significant increase, compared with the control animals, of 1.68 ± 0.12; p>0.01; n = 6).

### TGFβ2

**Fig 7:**
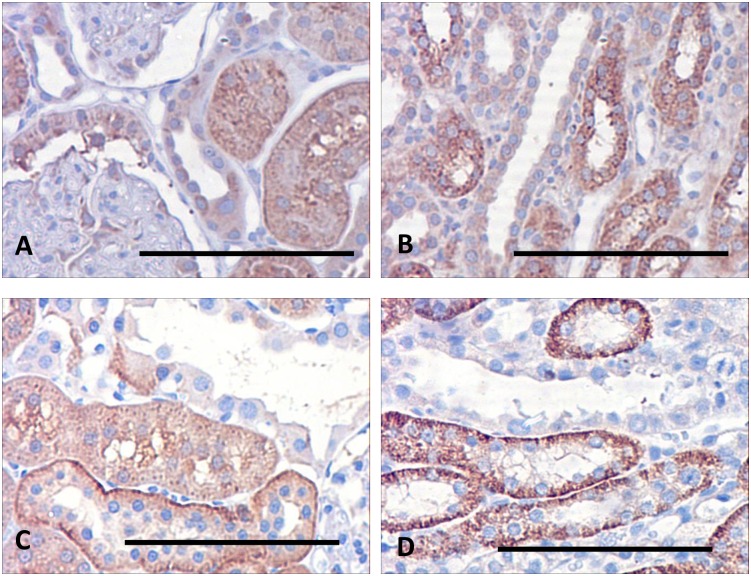
Immunohistochemical location of TGFβ2 in the kidneys of control rats and rats fed a lithium-containing diet for 6 months. (A) Cortical region of a control rat, showing weak cytoplasmic staining in the proximal tubules but an absence of stain in the distal tubule. (B) In the outer medulla, the collecting duct is unstained but there is some staining in the thick ascending limbs. (C) In the lithium-treated animals there is strong staining in the basolateral regions of the distal tubules, but no staining in the cortical collecting ducts. (D) This pattern is repeated in the outer medulla, where the thick ascending limbs show strong basolateral staining but the collecting duct is devoid of stain. Scale bar, 50 microns.

Fig 7A and 7B illustrates that in the control animals there is diffuse staining in the proximal tubules, and basolaterally in the distal tubules, while the cortical collecting ducts themselves are unstained. After adjustment for background staining, the area of cortical DAB stain was 2.88 ± 1.0%, while in the outer medulla it was slightly less at 1.98 ± 0.28%. In contrast, while cortical regions of the lithium-treated kidneys show little change in staining intensity (Fig 7C), there is a substantial increase in staining in the outer medulla, confined to the thick ascending limbs (Fig 7D). In these kidneys, the cortical area occupied by DAB staining was 6.54 ± 1.04% while the outer medullary area showed a very significant increase, compared to the controls, at 11.29 ± 1.2%. This difference is underlined by the qPCR results, which indicated that the presence of TGFβ2 in the cortex in lithium-treated rats increased by a factor of 1.23 ± 0.05; P>0.01; n = 6) while the increase in the medulla was considerable larger (1.45 ± 0.06; p>0.01; n = 6), which is reflected in the microarray data.

## Discussion

The present work is aimed primarily at the refinement of the diagnosis of kidney fibrosis and the investigation of potential markers and mechanisms of this disorder across species. In this respect, a MARGS-based fibrosis classifier, that is able to separate fibrotic from non-fibrotic tissues across different etiologies and solid organs of humans, including the kidney, has been developed [3, 5]. In this study it was extended to include a rat model of CKD. The MARGS-based fibrosis classifier gene set was able to classify all samples of medulla and cortex correctly with a classification rate, sensitivity and specificity of 100%. Using gene set enrichment analysis, which focused on 5 pathway gene sets known to be involved in fibrosis and ECM remodeling, the data support the more cortical pattern of fibrosis due to lithium exposure. This reflects previous reports in this disease model, since gene up-regulation in the cortex appears to be more consistent and less variable than in the medulla [8].

The microarray data of selected genes such as TGFβ2, CD44, and NOV/CCN3 were validated against immunohistochemistry and qPCR.

The successful application of this classifier to experimental lithium-induced interstitial fibrosis has significant consequences. First, these results argue for universal features in the pathophysiology of fibrosis across species. Second, it supports the use of this experimental model [8] for interventional studies with respect to the application of such findings in humans.

A chronic model of lithium-induced renal fibrosis, in rats, with only minimal active inflammation, mimicking many aspects of chronic interstitial fibrosis in human kidneys, has been described [8]. These rats exhibited progressive focal interstitial fibrosis, characterized by increased numbers of myofibroblasts, apparently enhanced TGFβ1 expression, and interstitial collagen deposition, as well as evidence of focal glomerulosclerosis [8]. However, exactly how lithium induces fibrosis is not well delineated.

In the past, transcriptome analyses of renal medulla from rats treated with lithium for 4 weeks, demonstrated altered transcription and mRNA expression of a number of genes, involved in cellular proliferation and regulation of the actin cytoskeleton [11–13]. NOV/CNN3 is a cysteine-rich protein that belongs to the CCN family of matricellular proteins interacting with integrins [14]. NOV is involved in extracellular matrix production, and migration of several cell types. It is widely expressed in several tissues, including the kidney [14, 15]. NOV differentially modulates expression of MMP-1, -3, -2, -9 and -13 in a cell-type specific manner [16–19]. NOV up-regulation in lithium-treated rats was associated with augmented MMP-2, CD44 and TGFβ2.

TGFβ is a potent inducer of ECM accumulation [20–23]. TGFβ1 and TGFβ2 are the major isoforms involved in renal fibrosis [24]. Upregulation of TGFβ has been associated with tubulointerstitial fibrosis in humans and in various experimental models of fibrosis [25]. Importantly, the therapeutic potential of an anti-TGFβ monoclonal antibody was demonstrated in rats with chronic renal allograft rejection [26], diabetes [27] and unilateral ureteral obstruction [28]. TGFβ1 is also known to regulate MMP expression, as shown previously [29].

CD44 is a transmembrane glycoprotein which occurs in a large number of functionally distinct isoforms, implicated in many physiological and pathological processes, including fibrosis [30]. Hyaluronic acid (HA) and osteopontin are major ligands of CD44. For example, upon binding with HA, CD44 interacts with TGFβ receptor I, resulting in the activation of TGFβ1 signaling in metastatic breast tumor cells [31]. Moreover, cleavage of pro-TGFβ1 into its active form by MMP-9 is dependent upon CD44 interaction with MMP-9 [32].

Glomerular or tubular CD44 overexpression occcurs in humans with IgA-nephropathy, diabetic nephropathy and hypertensive nephrosclerosis as well as in experimental mesangial proliferative glomerulonephritis [33–35]. In human IgA nephropathy and in renal allografts, tubular CD44 expression was found to correlate with IF/TA [36, 37]. During unilateral ureteral obstruction, CD44-deficient mice exhibited increased tubular damage, associated with decreased proliferation and increased apoptosis of tubular epithelial cells but decreased renal fibrosis [38]. The authors suggest that CD44, by interacting with TGFβ1, plays a signal role in the development of interstitial fibrosis.

MMP-2 degrades gelatin and many forms of collagen including collagen IV. It has been implicated in renal fibrosis, and renal tubular epithelial cell transdifferentiation with tubular atrophy. MMPs, including MMP-2 were shown to be increased in human and rat chronic allograft dysfunction [39]. Urinary MMP-2 and MMP-9 and TIMP-1 and TIMP-2 might be markers of renal allograft function in humans; especially increased MMP-9 concentrations were shown to correlate with histologic lesions related to tubular atrophy and interstitial fibrosis [40]. Accordingly, in humans with chronic transplant nephropathy with interstitial fibrosis, serum MMP-2 levels were found to be elevated compared to subjects with acute rejection or stable graft function [41]. In addition, a clear association between MMP-2 transcript abundance and the extent of tubular injury in human delayed graft function was observed recently [42]. In human renal allograft biopsies, augmented concentrations of MMP-7 and MMP-2 in serum were parallel to the degree of IF/TA [43,44,45]. In a Fischer-Lewis rat model of kidney transplantation, recipients initially develop acute rejection which subsequently leads to chronic renal allograft rejection associated with IF/TA [39]. During chronic renal allograft rejection, mRNA levels of MMPs (MMP-2, -11,-12, -14), TIMP-1,-2, ADAM-17 and TGFβ1 were increased [38]. In the present study, MMP-2 mRNA levels were found to be overexpressed in both the cortex and medulla of lithium-fed rats compared to controls.

In conclusion, we have demonstrated that a MARGS-based fibrosis classifier that can be applied successfully across solid organs in humans, can also be applied to the rat kidney. Thus the MARGS-based fibrosis classifier can be used as a valuable tool in the investigation of rat models of renal fibrosis to correlate with changes demonstrated in human renal fibrosis.
